# Insertion polymorphisms of *SINE200 *retrotransposons within speciation islands of *Anopheles gambiae *molecular forms

**DOI:** 10.1186/1475-2875-7-163

**Published:** 2008-08-25

**Authors:** Federica Santolamazza, Emiliano Mancini, Frédéric Simard, Yumin Qi, Zhijian Tu, Alessandra della Torre

**Affiliations:** 1 Istituto Pasteur-Fondazione Cenci-Bolognetti, Sezione di Parassitologia, Dipartimento di Scienze di Sanità Pubblica, Università di Roma "La Sapienza", Italy; 2 Institut de Recherche pour le Développement, UR016, and Institut de Recherche en Sciences de la Santé, Bobo-Dioulasso, Burkina Faso; 3 Department of Biochemistry, Virginia Polytechnic Institute and State University, Blacksburg VA, USA

## Abstract

**Background:**

SINEs (Short INterspersed Elements) are homoplasy-free and co-dominant genetic markers which are considered to represent useful tools for population genetic studies, and could help clarifying the speciation processes ongoing within the major malaria vector in Africa, *Anopheles gambiae *s.s. Here, we report the results of the analysis of the insertion polymorphism of a nearly 200 bp-long SINE (*SINE200*) within genome areas of high differentiation (i.e. "speciation islands") of M and S *A. gambiae *molecular forms.

**Methods:**

A *SINE*-PCR approach was carried out on thirteen *SINE200 *insertions in M and S females collected along the whole range of distribution of *A. gambiae *s.s. in sub-Saharan Africa. Ten specimens each for *Anopheles arabiensis*, *Anopheles melas, Anopheles quadriannulatus *A and 15 M/S hybrids from laboratory crosses were also analysed.

**Results:**

Eight loci were successfully amplified and were found to be specific for *A. gambiae *s.s.: 5 on 2L chromosome and one on X chromosome resulted monomorphic, while two loci positioned respectively on 2R (i.e. *S200 *2R12D) and X (i.e. *S200 *X6.1) chromosomes were found to be polymorphic. *S200 *2R12D was homozygote for the insertion in most S-form samples, while intermediate levels of polymorphism were shown in M-form, resulting in an overall high degree of genetic differentiation between molecular forms (Fst = 0.46 p < 0.001) and within M-form (Fst = 0.46 p < 0.001). The insertion of *S200 *X6.1 was found to be fixed in all M- and absent in all S-specimens. This led to develop a novel easy-to-use PCR approach to straightforwardly identify *A. gambiae *molecular forms. This novel approach allows to overcome the constraints associated with markers on the rDNA region commonly used for M and S identification. In fact, it is based on a single copy and irreversible *SINE200 *insertion and, thus, is not subjected to peculiar evolutionary patterns affecting rDNA markers, e.g. incomplete homogenization of the arrays through concerted evolution and/or mixtures of M and S IGS-sequences among the arrays of single chromatids.

**Conclusion:**

The approach utilized allowed to develop new easy-to-use co-dominant markers for the analysis of genetic differentiation between M and S-forms and opens new perspectives in the study of the speciation process ongoing within *A. gambiae*.

## Background

*Anopheles gambiae *sensu stricto (s.s.) is the most important vector of human malaria in Africa, causing 90% of the fatalcases worldwide [1]. It is believed that the differentiation of this very synanthropic and anthropophilic species within the *A. gambiae *complex is very recent, having taken place a few thousand years ago, as a result of expansion of human activities, which provided mosquitoes with new opportunities for breeding, eventually creating a worsening in malaria transmission in sub-Saharan Africa [2].

Chromosomal and molecular evidence from West Africa suggests that *A. gambiae *s.s. is currently undergoing incipient speciation leading to a segregation by reproductive isolation of (at least) two "molecular forms" provisionally named M and S [3-6]. These forms have a largely overlapping range west of the Great Rift Valley, although their relative frequencies are very different on a micro-geographic scale, probably due to adaptation to differentiated larval habitats [7-10]. Due to common background of shared ancestral polymorphisms and to the still ongoing (although limited) gene flow, M and S forms are characterized by an overall very low degree of genetic differentiation, which has been shown to be mostly restricted to three unlinked regions of their genome. Two are adjacent to the centromere of 2L and X chromosomes and the other is in a small portion of the 2R chromosome ("genomic islands of speciation" [11,12]). Although the overall picture suggests that we are observing speciation at its very early stages, the taxonomic status of *A. gambiae *s.s. molecular forms has not yet been established, nor has consensus been reached on whether or not they should be considered as entities on independent evolutionary trajectories, i.e. either as polymorphic components of a single species, or as emerging species. This issue is of great interest not only from an evolutionary point of view, but also because it has important implications both for malaria epidemiology and for the optimization of vector-based control strategies.

One major constraint to progress toward a solution of this debate is represented by difficulties in finding molecular markers with different/contrasting evolutionary dynamics, which would allow to get a better understanding of the strength of the reproductive barrier between molecular forms. In fact, so far, M and S forms are characterized by form-specific single nucleotide polymorphisms (SNPs) in the spacer regions of ribosomal DNA (rDNA) [13-15] and their population genetics has been analysed mostly by microsatellite approach, which present important intrinsic (e.g. low differentiation between M and S and homoplasy) and technical (e.g. need of sequencing facilities) drawbacks, which have limited their exploitation [16-19].

Recently, the analysis of the insertion patterns of transposable elements (TEs) (i.e. mobile genetic units capable of replicating and spreading in the host genome) has been successfully applied to support genetic differentiation between *A. gambiae *molecular forms [5,20-22]. Among TEs, Short INterspersed Elements (SINEs) have been extensively used as phylogenetic and population genetic markers in primate taxa [23] and, preliminary, in *A. gambiae *[5,20]. SINEs are 100–500 bp long non-autonomous retrotransposons occurring in large copy numbers in eukaryotic genomes [24-27], that need to recruit enzymes encoded by Long INterspersed Elements (LINEs) to mobilize after transcription via RNA polymerase III [28,29]. They present unique features absent in most other TEs, which make them particularly useful for phylogenetic and population genetic studies: i) they can be considered 'homoplasy-free characters' because the chance of independent insertions/excisions into/from the same site is remote; therefore, the ancestral state is represented by the absence of the element at a locus and shared insertions at that locus are identical by descent [23,30]; ii) since they are short, they can be amplified even from low-quality genomic DNA and insertion polymorphisms at individual genomic locations can be easily and rapidly assayed by PCR [31]; iii) polymorphic SINEs are believed to be recently inserted and, thus, can help illuminate recent evolutionary events and resolve complexities in the population genetics structure [30-34].

*SINE200 *is a ~200 bp element that is highly repetitive (>3,000 copies) and widespread in the *A. gambiae *s.s. genome [35]. Here we report the structure of this element and the results of a large scale analysis aimed to highlight different patterns of *SINE200 *insertion polymorphism between *A. gambiae *molecular forms at loci inside the speciation islands and propose the exploitation of these elements as novel molecular markers for the identification and/or population genetic analysis of M and S forms.

## Materials and methods

### *Anopheles gambiae *samples

**Figure 1:**
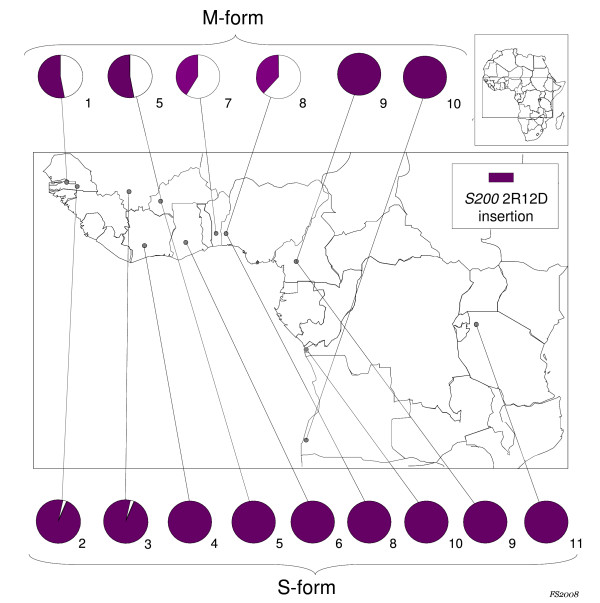
**Insertion frequencies at *S200 *2R12D locus in *Anopheles gambiae *M-form and S-form samples**. Numbers below the pie-charts refer to the sampling sites as listed in Table 1.

The study was carried out on *A. gambiae *s.s. M- and S-form adults collected between 1998 and 2006 in 11 African Countries (Figure 1, Table 1). Ten specimens of other species of *A. gambiae *complex, i.e. *A. arabiensis *from Senegal and Zimbabwe [5,36], *A. melas *from Angola [10] and *A. quadriannulatus *A from Zimbabwe [36] were also analysed.

**Table 1:** Insertion polymorphisms at loci *S200 *X6.1 and *S200 *2R12D in *Anopheles gambiae *molecular forms.

	**Country**	**Site**	**Form**	**N**	***S200 *X6.1**		***S200 *2R12D**	
					
					**H**	**AF**	**H**	**AF**
1	The Gambia	Maccarthy Island	M	15	0.00	1.00	0.40	0.53
2	Senegal	Kedougou	S	19	0.00	0.00	0.05	0.97
3	Mali	Banambani	S	32	0.00	0.00	0.00	0.98
			M	3	0.00	1.00	0.33	0.50
4	Ghana	Accra area	S	28	0.00	0.00	0.00	1.00
5	Burkina Faso	Bobo Dioulasso	S	27	0.00	0.00	0.00	1.00
			M	30	0.00	1.00	0.33	0.53
6	Ivory Coast	Buakè area	S	20	0.00	0.00	0.00	1.00
7	Benin	Dassa area	M	33	0.00	1.00	0.24	0.41
8	Nigeria	Kobape, Olugbo	S	20	0.00	0.00	0.00	1.00
			M	14	0.00	1.00	0.21	0.39
9	Cameroon	Mangoum	S	30	0.00	0.00	0.00	1.00
		Kribi	M	30	0.00	1.00	0.00	1.00
10	Angola	Cabinda	S	43	0.00	0.00	0.00	1.00
		Luanda area	M	16	0.00	1.00	0.00	1.00
11	Tanzania	Nyakariro, Kwagole	S	26	0.00	0.00	0.00	1.00

Sampling sites, number of specimens of *A. gambiae *molecular form analysed (N), heterozygosity (H) and allele frequency (AF). Sampling sites are listed from west to east and numbered as in Figure 1 (for information on the samples and sampling sites see della Torre *et al *[5]).

A cross between females of the GA-CAM (a M-form colony originated from field gravid females collected in Cameroon) and males of the GA-BF-5.7 colonies (a S-form colony originated from a single field gravid female collected in Burkina Faso) was performed and parental individuals and F1 hybrid females were analysed.

### Construction of *SINE200 *consensus sequence and copy number determination

*SINE200 *was first reported as part of the *A. gambiae *genome annotation [35]. Ninety-two *SINE200 *copies, which are 150 bp or longer, were randomly selected from the PEST genome (version P3, ). Alignment was performed using ClustalX with gap open penalty = 10, gap extension penalty = 0.05 [37]. The alignment was used as input for the program Consensus  and a *SINE200 *consensus sequence was created using majority rule. At three positions, where there was no simple majority base, manual inspection allowed us to assign ambiguous bases (e.g., W for A or T). The *A. gambiae *genome database was then searched by BLAST using the above mentioned consensus as a query and the e-value cutoff was set at e-10. BLAST hits shorter than 150 bp were not counted.

### Analysis of *SINE200 *insertion polymorphisms

Genomic DNA was extracted with various standard procedures, and specimens were identified to species and molecular forms by PCR-RFLP [38,39]. *SINE200 *elements were located *in silico *by BLASTN searches on the genome sequence of the *A. gambiae *PEST genome using the obtained *SINE200 *consensus sequence as a query. Thirteen *SINE200 *insertions lying within the *A. gambiae *molecular form speciation islands (*sensu *Turner [11]) on X, 2L and 2R chromosomes, and characterized by the presence of 500 bp flanking regions showing a single hit in the genome, were selected. Primers were designed to amplify across the element using Primer 3 software [40]. The selected loci were named '*S200' *followed by the abbreviation of the chromosomal arm (2L, 2R, X), by a number/letter corresponding to the chromosomal location on the cytogenetic map [4] and by an additional number aimed to distinguish primer sets positioned on the same chromosome division. Genes annotated within a 20 Kb genome sequence including *SINE200 *insertions for each locus were retrieved from the PEST genome ver. Agam P3 Feb. 2006 (Table 2).

**Table 2:** *SINE200 *primer list.

**Locus**	***SINE200 *coordinate**	**Primer pair (forward/reverse)**	**Product size (bp)**	**Annotated genes (in 20 Kb)**
***S200 *X6.1**	Chromosome X:	5'-TCGCCTTAGACCTTGCGTTA-3'	479	AGAP001076
	22951445–22951671	5'-CGCTTCAAGAATTCGAGATAC-3'		(CYP4G16 gene)
***S200 *X6.2**	Chromosome X:	5'-TCGGGCCAATATAACACAC-3'	588	AGAP001094
	24225524–24225731	5'-AGGCGCCATGTACGTAACC-3'		
***S200 *2L20A.1**	Chromosome 2L:	5'-TGCCCTGTTCAAGATTTCAT-3'	564	none
	641051–641259	5'-GGTCACTCACGCATACCGTCT-3'		
***S200 *2L20A.2**	Chromosome 2L:	5'-ACGCCAGACGGTTTCATATC-3'	611	none
	977287–977497	5'-CCTATCTTTAATTTATATTCGC-3'		
***S200 *2L20B.1**	Chromosome 2L:	5'-AACCTTACAATACACAAGAAC-3'	495	AGAP004725
	2796669–2796889	5'-CAGGAAAACGACTACTCGAAC-3'		AGAP004726
***S200 *2L20B.2**	Chromosome 2L:	5'-CGCGTTGATTAATAATCCCAC-3'	483	none
	1191908–1192118	5'-CCAGTCTCTGGACATGCTG-3'		
***S200 *2L20B.3**	Chromosome 2L:	5'-TTATCTGCGCGTGAGTGG-3'	515	Intron of AGAP004691
	1276754–1276921	5'-ATACCGCCTAAACGCATG-3'		(LIM gene)
***S200 *2R12D**	Chromosome 2R:	5'-AGAATGAATTGTATGGAACAGG-3'	535	AGAP002640
	24868905–24869106	5'-CTATTAAATGTGTCTCGCTCG-3'		(GPR-OR38 gene)

*SINE200 *locus names, chromosomal locations and coordinates, PCR primers, 'filled' PCR product sizes and annotated genes within 20 Kb including the *SINE200 *loci investigated are indicated by Ensemble Gene ID, gene names are in brackets.

PCR reactions were carried out in a 25 μl reaction which contained 1 pmol of each primer, 0.2 mM of each dNTP, 1.5 mM MgCl2, 2.5 U Taq polymerase, and 0.5 μl of template DNA extracted from a single mosquito. Thermocycler conditions were 94°C for 10 min followed by thirty-five cycles of 94°C for 30 s, 54°C for 30 s and 72°C for 1 min., with a final elongation at 72°C for 10 min, and a 4°C hold. The resulting products were analysed on 1.5% agarose gels stained with ethidium bromide, with low and high molecular weight bands corresponding to fragments containing or lacking the targeted *SINE200*, respectively.

PCR products representing 'filled' and 'empty' sites of *S200 *X6.1 locus on X chromosome were sequenced on both strands using ABI Big Dye Terminator v.2 chemistry and an ABI Prism 3700 DNA Analyser. Multiple alignments were performed using ClustalX [37]. All sequences were deposited in GenBank under accession numbers EU881868–EU881887.

Indices of polymorphism (i.e. *SINE200 *insertion frequency and heterozygosity) and differentiation (*Fst*) at polymorphic loci were computed using *Fstat *2.9.3.2 [41]. Significance was tested with Bonferroni-adjusted *P*-values, using the randomization approach implemented in *Fstat*.

## Results

### Structural features and chromosomal density of *SINE200 *in the *A. gambiae *genome

**Figure 2:**
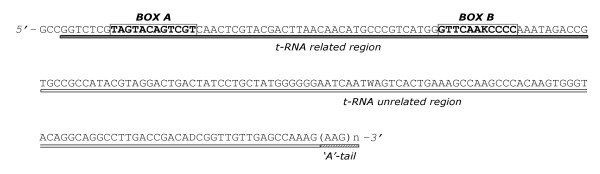
**Consensus sequence of *SINE200 *in *Anopheles gambiae *s.s**. *SINE200 *has a typical structure, with a tRNA-related sequence at its 5' end, a conserved tRNA-unrelated sequence, and simple repeats ('A' tail or tandem repeats) at its 3' end. The 5' end of the consensus (gray underlined) is 94% identical to the 5' end of a tRNA-Pseudo gene (AGAP000459). Sequences similar to the conserved A and B motifs for the polymerase III promoter are boxed. D:A/G/T; K:G/T; W: A/T.

*SINE200 *is a previously discovered *SINE *family of the *A. gambiae *genome [35]. Here we further characterized *SINE200 *by constructing a consensus sequence on the basis of 92 *SINE200 *copies that are 150 bp or longer, which is a small sample of all *SINE200 *copies (Figure 2). Analysis of the consensus sequence suggests that *SINE200 *has a typical structure, with a tRNA-related sequence at its 5' end, a conserved tRNA-unrelated sequence, and a simple repeat at its 3' end. Approximately 70 bp of the 5' end of the *SINE200 *consensus is 94% identical to the 5' end of a tRNA-Pseudo gene (AGAP000459). Sequences similar to the conserved A and B motifs for the polymerase III promoter were also found. Using the consensus sequence as a query, we showed that there are approximately 3,200 ubiquitous copies of *SINE200 *that are 150 bp or longer, and their density along the five chromosome arms ranges from 9.9 copies per Mbp (2R) to 12.9 copies per Mbp (X) (y3).

**Table 3:** *SINE200 *copy number and density on different chromosomes in *Anopheles gambiae *s.s.

**Chromosome Arm**	**Length (Mbp)**	***SINE200 *Copy No.**	**SINE200 Density (per Mbp)**
X	24,393	314	12,9
2L	49,364	520	10,5
2R	61,545	610	9,9
3L	41,963	529	12,6
3R	53,201	626	11,8
UNKNOWN	ND	615	ND

Copy number was determined by BLAST analysis using *SINE200 *consensus as query. The e-value cutoff is e-10. Only copies >150 bp are counted. ND = not determined.

### Analysis of *SINE200 *insertion polymorphism

The approach utilized was to design specific primers pair in the flanking regions of *SINE200 *insertions within M and S *A. gambiae *speciation islands, where a higher degree of form-specific genetic differentiation was expected. Although *SINE200 *are present in several copies also in the target regions, the selection of the loci has been more complicated than expected, mainly due to abundance of repetitive sequences in heterochromatic regions in centromeric areas of *A. gambiae *genome [35]. Eventually, 13 primer pairs were initially designed. Among these, 5 did not successfully amplified the targeted *SINE *insertions, as they did not yield bands or provided aspecific PCR products, and the analysis was therefore focused on the remaining 8 loci. Table 2 reports chromosomal location and annotated genes retrieved in the neighbouring genome areas of the 8 successfully amplified *SINE *loci. Each of these loci was initially scored for *SINE200 *insertion polymorphism by PCR-amplifying 15 M-form and 15 S-form specimens from either Burkina Faso and Cameroon and 15 S-form from Mali. *SINE200 *element insertions were found fixed in both forms in all five loci on the 2L speciation island, polymorphic in two loci positioned on 2R (i.e. *S200 *2R12D) and X (i.e. *S200 *X6.1) chromosomes, respectively, whereas a second *SINE200 *on centromeric area of X chromosome (*S200 *X6.2) was absent in all individuals analysed. *SINE200 *insertions were absent in all eight loci in the other analysed species of the *A. gambiae *complex (i.e. *A. arabiensis*, *A. melas *and *A. quadriannulatus *A).

The two polymorphic loci positioned on X and 2R chromosomes, were further studied by analysing additional 111 M-form and 200 S-form specimens (Table 1). In the case of *S200 *2R12D locus, all S-form specimens resulted homozygotes for the insertion, except for few individuals from Mali (allele frequency [AF] = 0.97) and Senegal (AF = 0.98), while intermediate levels of polymorphisms were shown in M-form (AF = 0.38–0.53), resulting in an overall high degree of genetic differentiation between molecular forms (*F*st = 0.46 P < 0.001). Moreover, preliminary results show intra-form differentiation between west (i.e. Burkina Faso, Nigeria and Benin) and west-central (i.e. Cameroon and Angola) M-subsamples (Fst = 0.46 P < 0.001), while no significant variation was found within each subsample. On the other hand, no intra-form differentiation at the same locus was recorded in the S-form sample within the same range of distribution.

**Figure 3:**
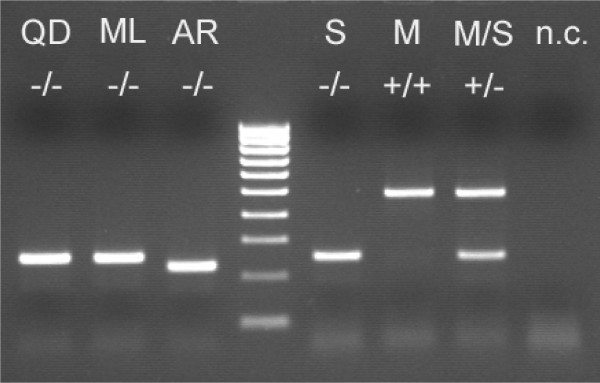
**Diagnostic PCR based on *S200 *X6.1 in *Anopheles gambiae *s.l**. PCR results from locus *S200 *X6.1 indicating the presence (+) or absence (-) of the insertion in females of *Anopheles gambiae *species complex. QD = *A. quadriannulatus *A; ML = *A. melas*; AR = *A. arabiensis*; S = *A. gambiae *S-form; M = *A. gambiae *M-form; M/S = M/S hybrids from laboratory crosses; n.c. = negative control. Ladder = 100 bp (BIOLINE HyperLadder IV).

**Figure 4:**
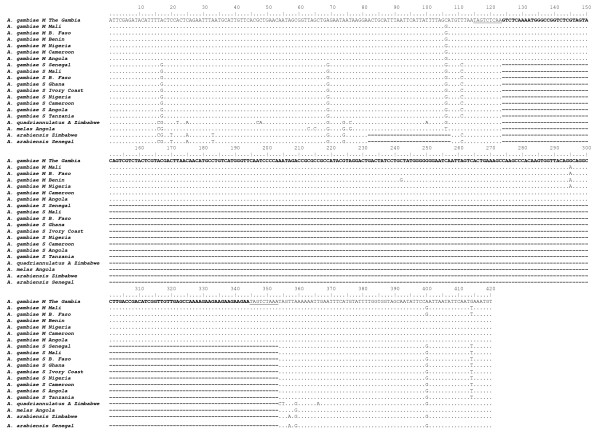
**Sequence alignment of *S200 *X6.1 and flanking regions in the *Anopheles gambiae *s.l**. Nucleotide substitutions at each position are indicated with the appropriate nucleotide. Deletions are denoted by dashes (-). The nucleotide deletion of 26 bp in the flanking region of *S200 *X6.1 for *A. arabiensis *corresponds to positions 82–107. Deletion at positions 124–353 corresponds to the absence of the *S200 *X6.1 element in *A. gambiae *S form, *A. quadriannulatus *A, *A. arabiensis *and *A. melas*. Target site duplications (TSDs) are underlined.

Remarkable differences among molecular forms were found at locus *S200 *X6.1 (Table 1): in all samples the insertion was fixed in M-form individuals, from which a single PCR product of 479 bp was amplified, and absent in S-form specimens, from which a 249 bp product was obtained (Figure 3). As expected, laboratory-reared specimens of both molecular forms analysed (N = 60) showed the same pattern of insertion at *S200 *X6.1 locus. Moreover, the analysis of M/S hybrids resulting from laboratory crosses produced consistent results, yielding both PCR-bands from all hybrid M/S specimens analysed (N = 15) (Figure 3). The absence of *SINE200 *at this locus was confirmed in all the other *A. gambiae *s.l. member species analysed. Interestingly, the PCR product obtained from *A. arabiensis *was represented by a 223 bp band, due to a 26 bp deletion in the *S200 *X6.1 flanking region. In addition, the alignment of *S200 *X6.1 flanking regions sequences showed (Figure 4): i) fixed mutations at 3 positions in the alignment (positions 16, 74, 359) differentiating both *A. gambiae *s.s. molecular forms from *A. arabiensis*, *A. melas *and *A. quadriannulatus*; ii) mutations in two positions (pos. 17, 69) differentiating the M-form from all the other taxa analysed; iii) a very high conservation in the sequence of the element was found in all M individuals analysed.

## Discussion

The analysis of the consensus sequence of *SINE200 *indicates that it is a typical tRNA-related SINE element. In fact, it has a tRNA-related region at the 5' end with the A and B boxes found in polymerase III promoters. It also has a variable number of the AAG tandem repeat at the 3' end, which is also typical for tRNA-related SINEs [42]. The middle of *SINE200 *is a conserved sequence that is not related to tRNA sequences, as already described for other eukaryotic *SINE *elements [43].

Eight *SINE200 *loci within *A. gambiae *s.s. speciation islands were analysed, as follows: i) two on the X-chromosome, one of which (i.e. *S200 *X6.2) was absent in all specimens tested, while the other (i.e. *S200 *X6.1) was fixed in the M-form and absent in the S-form samples; ii) one on 2R (i.e. *S200 *2R12D), which was found polymorphic in both molecular forms; and iii) five on 2L, which were all fixed in both forms. The observed high frequency of fixation of the insertions in centromeric areas probably reflects a common behaviour of transposable elements, which tend to accumulate in regions of reduced recombination [44], as also suggested for other retrotrasposon classes in the *A. gambiae *genome [21].

The observed differences in the allelic frequencies at *S200 *2R12D locus highlight a significant reduction of gene-flow between the two molecular forms. This represents an additional evidence in support of the relevance of this small chromosomal region in the speciation process ongoing within *A. gambiae *s.s., as proposed by Turner *et al *[11]. Interestingly, *S200 *2R12D lies in close proximity (about 20 Kb) to an odour receptor gene (i.e. GPR-OR38), which has been suggested to be likely related to reproductive isolation between molecular forms [12]. Moreover, a similar level of differentiation was observed within M-form, suggesting a subdivision between western and western-central M-populations (Figure 1). This sub-structuring observed within the M-form is consistent with recent evidence from a wide microsatellite analysis carried out on the same M-form populations [45] and with previous observations by Slotman *et al *[6], who suggests that M populations from Mali and Cameroon may no longer be considered a "single entity". It should be noted, however, that *S200 *2R12D locus lies within 2Rb chromosomal inversion, which is shared by M and S forms and shows different frequencies in various eco-geographic areas [4,5]. It is thus possible that the spread of this element in natural populations is affected by 2Rb inversion polymorphism, although preliminary data show that *S200 *2R12D insertion is not exclusive of one of the two alternative chromosomal arrangements (i.e. 2R+^b ^and 2Rb). Further studies on larger karyotyped samples are ongoing to evaluate a possible association between the 2Rb inversion and the element insertion.

As it is recognized that *SINE*s do not excide from a genome after their insertion [23,30] and since all *SINE200 *loci analysed were found to be specific of *A. gambiae *s.s., the analysed insertions likely occurred after divergence of this species from the other members of the *A. gambiae *complex. Moreover, *S200 *X6.1 was found to be exclusive of and highly conserved in the M-form and, therefore, probably recently integrated in its genome after divergence of molecular forms within the chromosome-X speciation island. This locus lies in proximity of CYP4G16, a gene of the cytochrome P450 family which has been indicated as a candidate gene in the incipient speciation process ongoing within *A. gambiae *s.s. [11].

In addition to the above cited indications in favour of a possible fruitful exploitation of *SINE200 *in the study of the sub-structuring of *A. gambiae*, the exclusive presence of *S200 *X6.1 in the M-form allows to propose a novel straightforward approach to distinguish *A. gambiae *s.s. molecular forms. In fact, all methods developed so far for their identification are based on point mutations in IGS region of rDNA, which is formed by several tandem arrays known to be subjected to concerted evolution. Thus, possible diagnostic problems, in particular in the interpretation of hybrid M/S patterns, may arise from incomplete homogenization of the arrays through concerted evolution and/or mixtures of M and S IGS-sequences among the arrays of single chromatids, due to recombination between copies on the X and Y chromosomes [15]. The *S200 *X6.1 locus, on the other hand, although located only about 1 Mb from IGS-region, does not show these constraints, being present in a single copy on the X-chromosome. Moreover, it is important to highlight that PCR-RFLP [38,39], and IMP-PCR [13,46] methods currently used for M and S identification are based on the recognition of single/few mutation(s), and thus subjected to homoplasy. On the other hand, the PCR diagnostic approach here proposed is based on the specific and irreversible insertion of a 230 bp element in the M-form (and its absence in S-form), thus allowing an unambiguous, simple and straightforward recognition of M and S forms (Figure 3). It is also interesting to note that, although the S-form amplicon is identical to those of *A. melas *and *A. quadriannulatus*, the 26 bp deletion reported for *A. arabiensis *allows to propose the use of the novel approach to discriminate *A. gambiae *from *A. arabiensis *specimens without preliminary species identification in large areas of sub-saharan Africa where *A. gambiae *molecular forms and *A. arabiensis *are the only species of the complex present.

## Conclusion

The approach utilized opens new perspectives in the studies of *A. gambiae *molecular forms. Further analyses on *SINE200 *loci mapping in different areas of *A. gambiae *genome are ongoing based on preliminary selection by a genome-wide TE-display approach of form-specific or polymorphic loci, to eventually provide additional, new efficient co-dominant markers for the analysis of genetic differentiation between M and S-forms.

## Competing interests

The authors declare that they have no competing interests.

## Authors' contributions

FS and EM carried out the molecular processing, participated in the analysis and interpretation of data, and in the drafting of the manuscript; YQ contributed to the molecular processing; FS collected part of the samples and to the drafting of the manuscript; ZT proposed the study and contributed to the set-up of the experimental approach, data analysis and drafting of the manuscript; AdT conceived and coordinated the study and wrote the manuscript. All authors read and approved the final manuscript.
